# The relative efficacy and safety of targeted agents used in combination with chemotherapy in treating patients with untreated advanced gastric cancer: a network meta-analysis

**DOI:** 10.18632/oncotarget.15923

**Published:** 2017-03-06

**Authors:** Shuping Xie, Huixiang Zhang, Xueyan Wang, Quanxing Ge, Junhong Hu

**Affiliations:** ^1^ Department of Gastroenterology, Huaihe Hospital of Henan University, Kaifeng, Henan, 475000, China; ^2^ Department of Radiotherapy, Huaihe Hospital of Henan University, Kaifeng, Henan, 475000, China; ^3^ Department of General Surgery, Huaihe Hospital of Henan University, Kaifeng, Henan, 475000, China

**Keywords:** gastric cancer, targeted therapy, efficacy and safety, network meta-analysis

## Abstract

Gastric cancer is one of the leading mortal causes. Targeted therapy is a new type of cancer treatment, which precisely identifies and attacks cancer cells and significantly reduces side effects. In this network meta-analysis, we focused on the efficacy and safety of 12 targeted agents on gastric cancer among a total of 8,405 patients from 24 trials. Hazard ratio (HR) with 95% credible interval (CrI) were calculated for primary outcomes, including overall survival (OS) and progression-free survival (PFS), while odds ratio (OR) with 95% CrI were calculated for secondary outcomes. Surface under the cumulative ranking curve (SUCRA) were calculated to illustrate the rank probability of various agents for different outcomes. Compared with other analyzed treatments, ramucirumab is outstanding in survival outcomes. However, higher risk of hematological events should be noted during its application. Lapatinib is also efficacious in progression reduction, while it is always combined with severe gastrointestinal events. Trastuzumab is proposed for its high efficacy in improving survival rate and safety, which is proper for most patients. In conclusion, trastuzumab was recommended as the optimal targeted agent combined with chemotherapy for gastric cancer patients.

## INTRODUCTION

Gastric cancer is one of the leading causes of death, ranking the fourth in the prevalence of cancer. Globally, 723,100 deaths and 951,600 new cases occur each year [1]. Meanwhile, gastric cancer takes the third place in the global burden of disability-adjusted life-years, following lung and liver cancers [2]. Thanks to the improvement of dietary, sanitation and development of antibiotics, the incidence rate and mortality of gastric cancer has decreased recently. However, in developing countries, it remains to be a great threat to our life, with the 5-year survival rate less than 25% [3]. In addition to environment factors, gastric cancer with a specific gene and family background is also prevailing.

Chemotherapy has been proved to be an effective treatment for patients with advanced gastric cancer, and currently, combination of fluoropyrimidine with platinum or irinotecan, triplet combination of fluoropyrimidine/platinum with docetaxel or epirubicin are provided as the standard first-line chemotherapy for patients with advanced gastric cancer [4–6]. However, it usually takes a few months to produce clinical therapeutic effect and patients often suffer from severe adverse effects including vomiting, nausea, rashes, headache, etc. Moreover, more than half of the patients may not response to the therapy [7]. To further improve the effect of chemotherapy and reduce corresponding toxicity, targeted therapy was introduced.

Targeted therapy is a new type of cancer treatment and a special type of chemotherapy. Agents used in targeted therapy can precisely identify and attack certain type of cancer cells based on the mutation of gene and protein. Meanwhile, little damage is done to normal cells, thus targeted therapy can significantly avoid side effects. Used in in combination with chemotherapy, targeted therapy is effective in many cancers. For example, Herceptin (trastuzumab), which targeted at breast cancers with the over-expression of Human Epidermal Growth Factor Receptor 2 (HER2), has been proved to significantly improve overall survival (OS) and disease-free survival (DFS) for both early-stage and advanced patients according to a 2014 Cochrane Review [8]. However, for patients with advanced gastric cancer, there is no standard targeted therapy, and this network meta-analysis was designed to evaluate the performance of different targeted drugs used in combination with chemotherapy and try to find out the most effective one/ones.

## RESULTS

### Study characteristics

**Figure 1:**
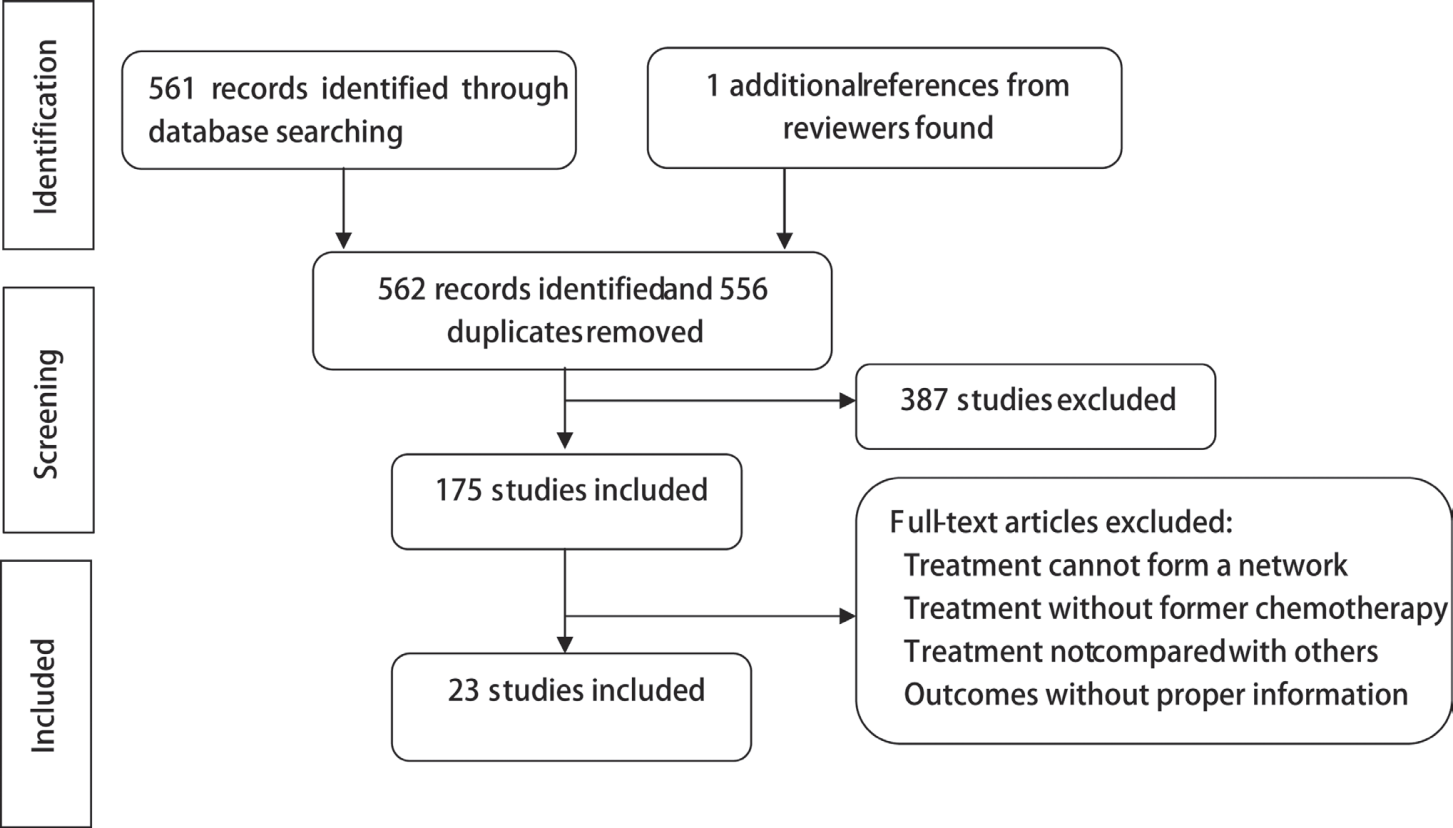
Flow chart of included studies

A total of 8,405 patients from 23 RCTs were enrolled in our analysis and listed in Supplementary Table 1 [9–31]. Twelve regimens on 7 targets were involved, including ramucirumab, bevacizumab, nimotuzumab, cetuximab, lapatinib, trastuzumab, endostar, everolimus, matuzumab, onartuzumab, panitumumab and sunitinib. The process of selection was illustrated in flow chart as Figure 1.

**Figure 2:**
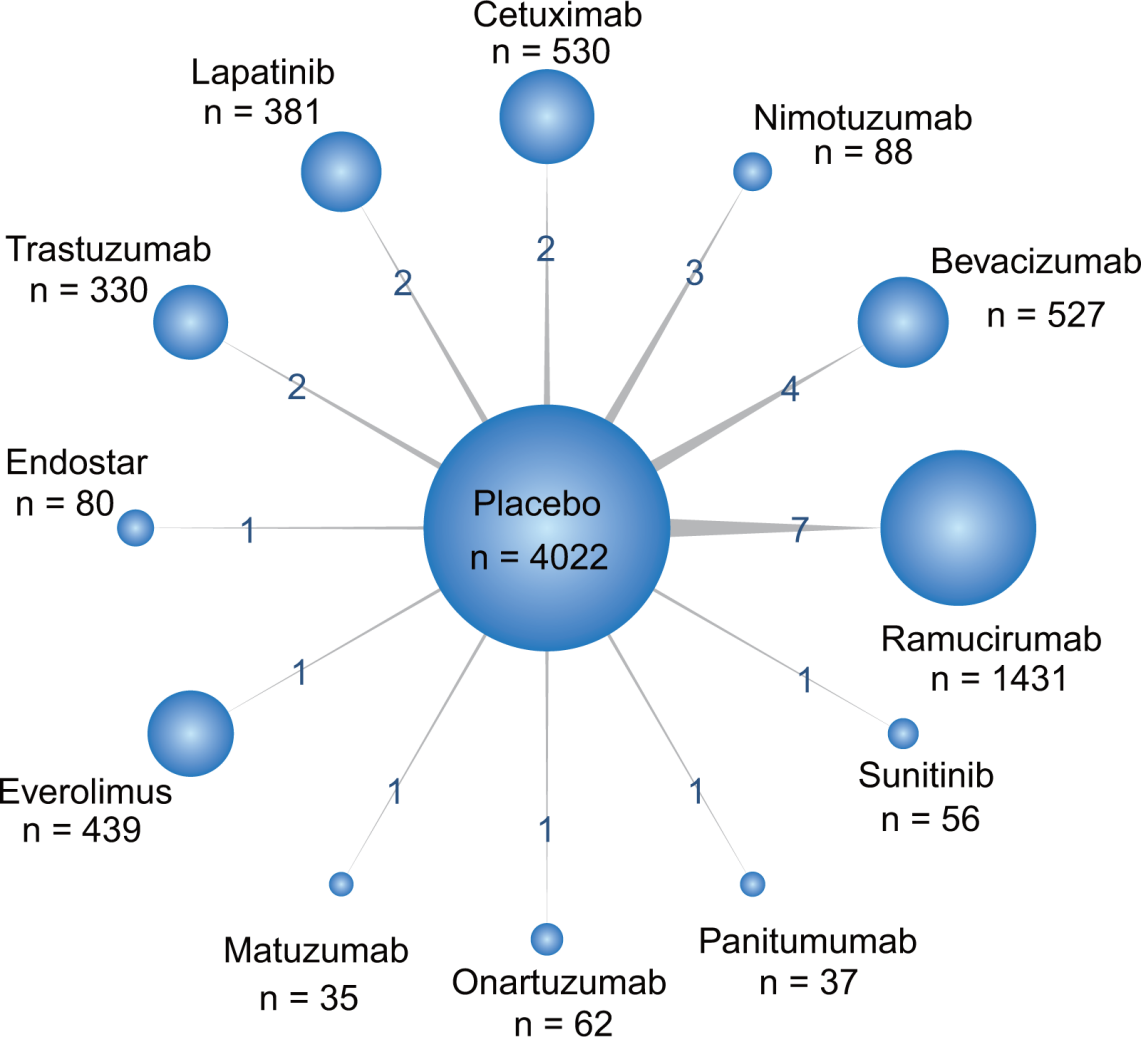
Network of all randomized controlled trials comparing primary outcomes of different targeted therapies for gastric cancer The width of the lines is proportional to the number of trials comparing each pair of treatments with numbers on the lines illustrating the exact number; the area of circles represents the cumulative number of patients for each intervention.

Network plot in Figure 2 presented the comparisons of various treatments for different outcomes. The width of the lines is proportional to the number of trials comparing each pair of treatments and numbers on the lines illustrate the exact number; the area of circles represents the cumulative number of patients for each intervention.

### Overall survival

**Figure 3:**
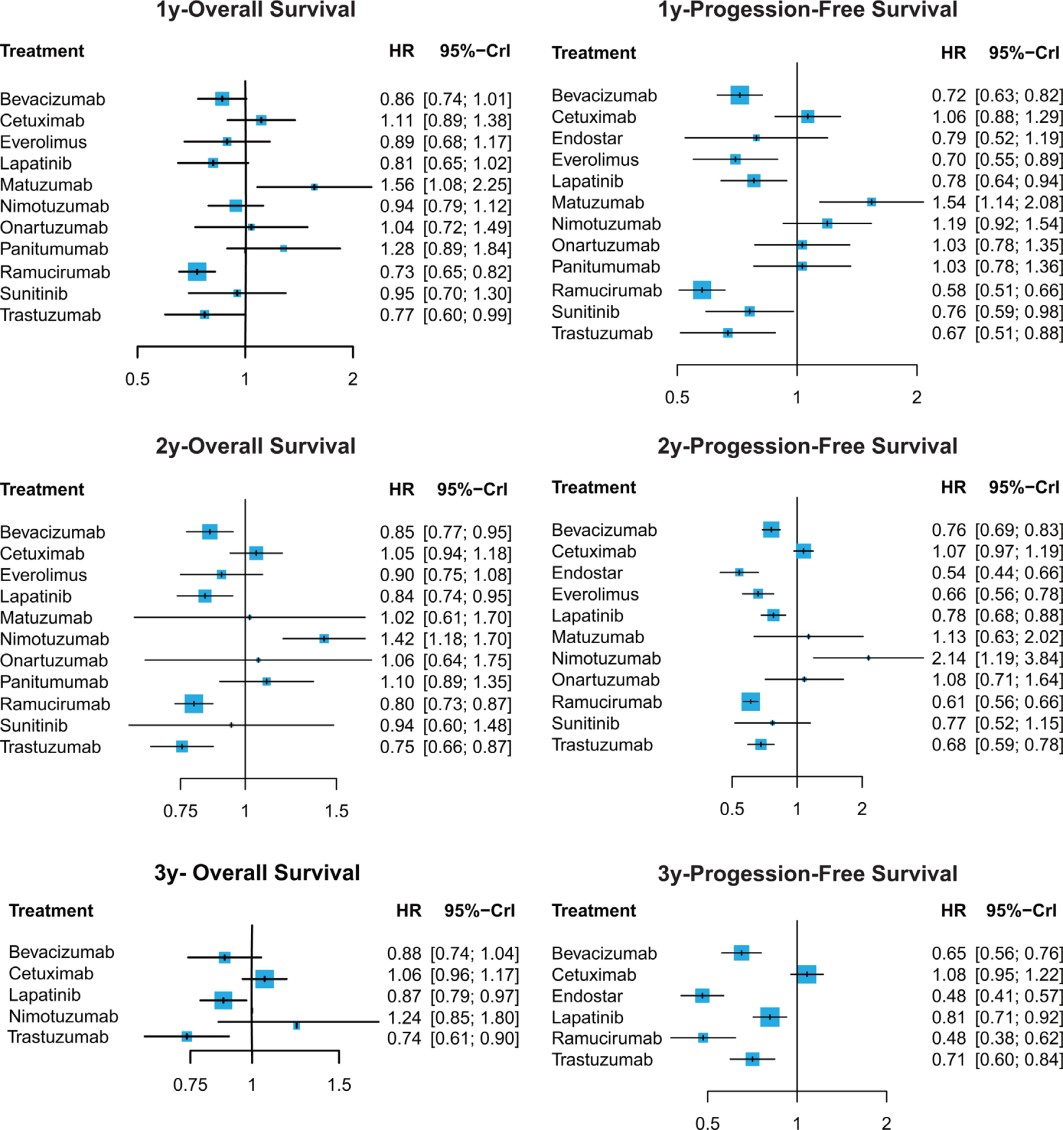
Hazard ratios (95% credible intervals) for network comparison of primary outcomes for gastric cancer treatments

**Figure 4:**
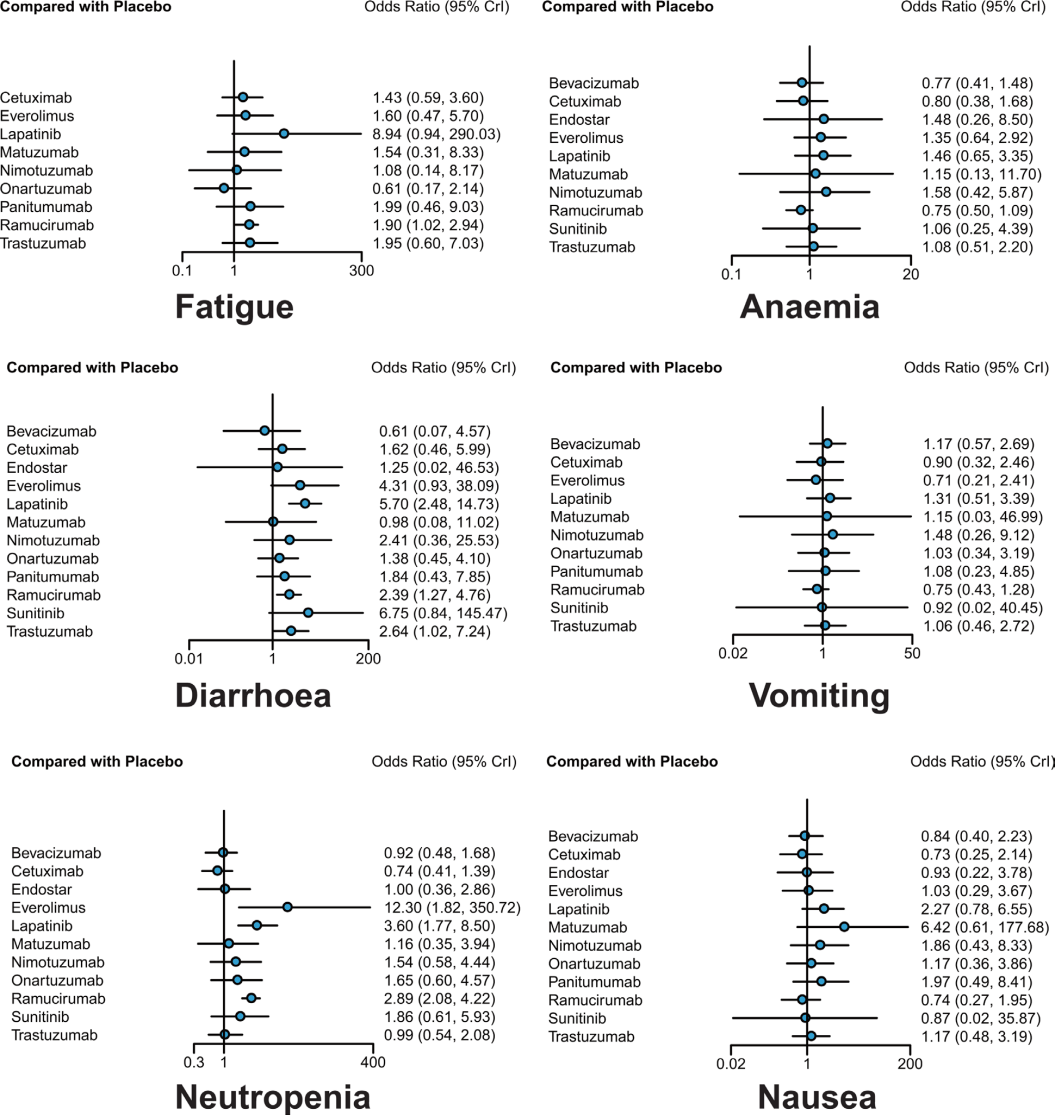
Odds ratios (95% credible intervals) for network comparison of secondary outcomes for gastric cancer treatments

The network meta-analysis results were listed in Supplementary Tables 2, 3 and Figures 3, 4. For 1y-OS, only trastuzumab and ramucirumab were found to be helpful compared to placebo (placebo vs. trastuzumab: HR = 1.30, 95% CrI: 1.01–1.67; placebo vs. ramucirumab: HR = 1.36, 95% CrI: 1.21–1.53). They were also superior than cetuximab, matuzumab and panitumumab (cetuximab vs. ramucirumab: HR = 1.51, 95% CrI: 1.18–1.93; cetuximab vs. trastuzumab: HR = 1.44, 95% CrI: 1.03–2.01; matuzumab vs. ramucirumab: HR = 2.13, 95% CrI: 1.45–3.12; matuzumab vs. trastuzumab: HR = 2.03, 95% CrI: 1.30–3.17; panitumumab vs. ramucirumab: HR = 1.74, 95% CrI: 1.19–2.55; panitumumab vs. trastuzumab: HR = 1.66, 95% CrI: 1.07–2.59). Besides, matuzumab was less efficacious than most of treatments including placebo. As for 2y-OS, bevacizumab, lapatinib, ramucirumab and trastuzumab were observed to perform better than placebo (bevacizumab vs. placebo: HR = 0.85, 95% CrI: 0.77–0.95; lapatinib vs. placebo: HR = 0.84, 95% CrI: 0.74–0.95; placebo vs. ramucirumab: HR = 1.26, 95% CrI: 1.15–1.37; placebo vs. trastuzumab: HR = 1.32, 95% CrI: 1.15–1.52). These four agents also performed better than cetuximab, nimotuzumab and panitumumab. Nimotuzumab showed worse efficacy than most treatments and placebo. Concerning 3y-OS, lapatinib was observed to have better perform than cetuximab (cetuximab vs. lapatinib: HR = 1.21, 95% CrI: 1.04–1.41). Trastuzumab showed a significantly better efficacy than cetuximab, nimotuzumab and placebo (cetuximab vs. trastuzumab: HR = 1.44, 95% CrI: 1.15–1.79; nimotuzumab vs. trastuzumab: HR = 1.68, 95% CrI: 1.10–2.57; placebo vs. trastuzumab: HR = 1.36, 95% CrI: 1.11–1.65).

### Progression-free survival

Bevacizumab, everolimus, lapatinib, ramucirumab, trastuzumab and sunitinib were found to be efficacious in improving 1y-PFS compared to placebo (bevacizumab vs. placebo: HR = 0.72, 95% CrI: 0.63–0.82; everolimus vs. placebo: HR = 0.70, 95% CrI: 0.55–0.89; lapatinib vs. placebo: HR = 0.78, 95% CrI: 0.64–0.94; placebo vs. ramucirumab: HR = 1.73, 95% CrI: 1.52–1.98; placebo vs. sunitinib: HR = 1.32, 95% CrI: 1.02–1.69; placebo vs. trastuzumab: HR = 1.49, 95% CrI: 1.13–1.96). Ramucirumab was more efficacious than major portion of the treatments, which was consistent with the result in OS. And everolimus showed a good efficacy in 1y-PFS, better than cetuximab, matuzumab, nimotuzumab, onartuzumab, panitumumab as well as placebo (cetuximab vs. everolimus: HR = 1.52, 95% CrI: 1.12–2.07; everolimus vs. matuzumab: HR = 0.46, 95% CrI: 0.31–0.67; everolimus vs. nimotuzumab: HR = 0.59, 95% CrI: 0.41–0.84; everolimus vs. ontaruzumab: HR = 0.68, 95% CrI: 0.47–0.98; everolimus vs. panitumumab: HR = 0.68, 95% CrI: 0.47–0.98; everolimus vs. placebo: HR = 0.70, 95% CrI: 0.55–0.89). However, matuzumab were not as efficacious as most treatments, meanwhile it was the only treatments performed worse than placebo. For 2y-PFS, nimotuzumab performed worse than all the treatments except matuzumab and onartumumab. Endostar had a better effect than bevacizumab, cetuximab, lapatinib, matuzumab, nimotuzumab, onartuzumab and placebo (bevacizumab vs. endostar: HR = 1.41, 95% CrI: 1.12–1.76; cetuximab vs. endostar: HR = 1.98, 95% CrI: 1.58–2.49; endostar vs. lapatinib: HR = 0.70, 95% CrI: 0.55–0.88; endostar vs. matuzumab: HR = 0.48, 95% CrI: 0.26–0.88; endostar vs. nimotuzumab: 0.25, 95% CrI: 0.14–0.47; endostar vs. onartuzumab: 0.50, 95% CrI: 0.31–0.79; endostar vs. placebo: 0.54, 95% CrI: 0.44–0.66). Moreover, ramucirumab were also superior to over half of the treatments. In 3y-PFS, both endostar and ramucirumab were significant helpful in progression reduced than other treatments. Cetuximab was less efficacious than any other treatments except placebo (bevacizumab vs. cetuximab: HR = 0.60, 95% CrI: 0.49–0.73; cetuximab vs. endostar: HR = 2.25, 95% CrI: 1.83–2.77; cetuximab vs. lapatinib: HR = 1.33, 95% CrI: 1.11–1.60; cetuximab vs. ramucirumab: HR = 2.24, 95% CrI: 1.69–2.96; cetuximab vs. trastuzumab: HR = 1.53, 95% CrI: 1.23–1.89).

### Adverse effect

With respect to fatigue, onartuzumab was reported to be associated with relatively low incidence rate compared to lapatinib (OR = 0.07, 95%CrI: 0.00–0.89), and ramucirumab was the only one performing worse than placebo (OR = 1.90, 95%CrI: 1.02–2.94).

In terms of neutropenia, patients treated with everolimus and lapatinib reported higher incidence rate than those treated with bevacizumab (OR = 13.60, 95%CrI: 1.80–399.41; OR = 3.90, 95%CrI: 1.57–11.82), cetuximab (OR = 16.61, 95%CrI: 2.20–497.70; OR = 4.85, 95%CrI: 1.92–13.60), endostar (OR = 12.55, 95%CrI: 1.42–407.48; OR = 3.63, 95%CrI: 14.15), trastuzumab (OR = 12.30, 95%CrI: 1.60–368.71; OR = 3.63, 95%CrI: 1.34–10.18), and even placebo (OR = 12.30, 95%CrI: 1.82–350.72; OR = 3.60, 95%CrI: 1.77–8.50, respectively). Ramucirumab yielded similar results, with higher risk of neutropenia compared with placebo (OR = 2.89, 95%CrI: 2.08–4.22), bevacizumab (OR = 3.10, 95%CrI: 1.60–6.75), cetuximab (OR = 3.90, 95%CrI: 1.92–7.85) and trastuzumab (OR = 2.92, 95%CrI: 1.31–5.93).

As for diarrhoea, some treatments performed worse than placebo, including lapatinib (OR = 5.70, 95% CrI: 2.48–14.73), ramucirumab (OR = 2.39, 95% CrI: 1.22–4.76), trastuzumab (OR = 2.64, 95%CrI: 1.02–7.24), while onartuzumab showed significant superiority to lapatinib (OR = 0.24, 95% CrI: 0.06–0.92), which was consistent with the result of fatigue.

In the meanwhile, no statistical difference was found in terms of anaemia, vomiting and nausea.

### Ranking analysis

To help us with a better understanding of the results, we plotted the cumulative ranking probability curves to calculate SUCRA value for all medications on investigated outcomes. As illustrated in Table 1, ramucirumab showed a high efficacy in all survival outcomes except for the lack of 3y-OS as well as trastuzumab was efficacious in all OS and 1y-PFS. Lapatinib was another good treatment for improving OS, while endostar and everolimus were effective agents for PFS items. For secondary outcomes, Disappointingly, almost all treatments ranked lower than placebo. However, exceptions existed. Cetuximab was associated with lower risk of neutropenia and nausea than all other treatments, ramucirumab exhibited better performance in terms of anaemia and vomiting compared to other treatments, while onartuzumab and bevacizumab were the effective treatment in controlling fatigue and diarrhoea respectively.

**Table 1:** SUCRA value of treatments for gastric cancer therapy

	1-OS	2-OS	3-OS	1-PFS	2-PFS	3-PFS	Fatigue	Anaemia	Neutropenia	Diarrhoea	Vomiting	Nausea
**Placebo**	0.384	0.387	0.364	0.331	0.300	0.148	0.747	0.523	0.694	0.785	0.502	0.588
**Bevacizumab**	0.673	0.681	0.683	0.735	0.561	0.630	-	0.726	0.746	0.819	0.400	0.674
**Cetuximab**	0.252	0.294	0.192	0.249	0.203	0.019	0.531	0.695	0.870	0.578	0.570	0.743
**Endostar**	-	-	-	0.609	0.972	0.919	-	0.377	0.686	0.608	-	0.611
**Everolimus**	0.607	0.585	-	0.761	0.767	-	0.474	0.331	0.036	0.253	0.675	0.570
**Lapatinib**	0.760	0.727	0.708	0.627	0.532	0.354	0.089	0.302	0.142	0.147	0.353	0.237
**Matuzumab**	0.036	0.415	-	0.015	0.238	-	0.501	0.478	0.611	0.691	0.475	0.115
**Nimotuzumab**	0.511	0.032	0.087	0.146	0.010	-	0.625	0.320	0.471	0.440	0.359	0.338
**Onartuzumab**	0.373	0.366	-	0.295	0.241	-	0.876	-	0.445	0.643	0.488	0.507
**Panitumumab**	0.148	0.248	-	0.295	-	-	0.392	-	-	0.531	0.476	0.306
**Ramucirumab**	0.923	0.835	-	0.969	0.881	0.909	0.377	0.769	0.202	0.424	0.706	0.734
**Sunitinib**	0.501	0.519	-	0.660	0.571	-	-	0.503	0.402	0.189	0.525	0.581
**Trastuzumab**	0.832	0.912	0.966	0.808	0.726	0.522	0.389	0.477	0.695	0.392	0.471	0.497

### Jadad scale

The Jadad Scale of included studies demonstrated in Supplementary Table 4 showed that all trails were randomized and controlled, but unfortunately, the majority of included studies were open-label trials and only a few adopted double-blinding methods, which might add to the heterogeneity of this NMA. Also, as was expected, there were withdrawals in all studies, partly due to the intensity of chemotherapy.

## DISCUSSION

In the current analysis, we assessed the efficacy and safety of common targeted agents used in combination with chemotherapy. including 12 regimens on 7 targets, i.e. HER2 (trastuzumab, lapatinib), EGFR (nimotuzumab, panitumumab, matuzumab, cetuximab), VEGF (bevacizumab, endostar), VEGFR (ramucirumab), TKI (sunitinib), HGFR(onartuzumab) and mTOR (everolimus).

Human epidermal growth factor receptor-2 (HER2) mutation is one prevailing mutation in the pathogenesis of gastric cancer [32]. Trastuzumab is a monoclonal anti-HER2 antibody, whereas lapatinib is a tyrosine kinase inhibitor of both ERB1 and HER2. For HER2 mutation, we found that both trastuzumab and lapatinib were effective in OS and trastuzumab was also good in short-term PFS, yet lapatinib had a higher risk of adverse events and trastuzumab exhibited mediocre performace in controlling all adverse events except neutropenia. Actually, trastuzumab has been reported to be effective in combination with first-line fluoropyrimidine and cisplatin therapy [33]. And the addition of trastuzumab to chemotherapy for gastric and gastroesophageal cancer significantly yielded survival benefits compared to other antibodies [34]. It was also reported that the association of trastuzumab with oxaliplatin and fluoropyrimidin could reduce the toxicity of therapy compared with the addition of fluoropyrimidin and cisplatin [35], which was consistent with the results of our NMA to some extent.

Vascular endothelial growth factor (VEGF) and its receptors (VEGFR) play critical roles in the angiogenesis and metastasis of gastric cancer [36]. Ramucirumab is a monoclonal anti-VEGFR-2 antibody and bevacizumab is the antibody of VEGF-A, both widely used in the treatment of lung, breast, and renal cancer. According to our study, ramucirumab performed extraordinarily well in all survival terms, which was consistent with the results of previously reported studies [37], and was associated with relative low risk of anaemia, neutropenia and vomiting, though the results with regard to fatigue, neutropenia and diarrhoea were not desirable. As for bevacizumab, although was not as effective as ramucirumab, still yielded relatively good results in improving survival rate while caused few adverse events according to this NMA. Yet according to previous RCTs, bevacizumab showed no significant benefit on improving survival rate [26, 38], so its performance need to be further evaluated in future studies.

Mutations of epidermal growth factor receptor (EGFR) contribute to the growth and metastasis of tumor. Nimotuzumab, panitumumab, matuzumab and cetuximab are all antibodies of EGFR. By inhibiting the over-expression of EGFR, they help to restrict the growth, invasion and metastasis of tumor. According to our results, the efficacy of anti-EGFR treatments was not superior to other therapies. Nimotuzumab and matuzumab were even less efficacious than placebo in some outcomes. Also there was one clinical report suggested that the addition of nimotuzumab weakened the antitumor effect of cisplatin regimen, which is the first line chemotherapy for patients, and this lead to a worse efficacy than the addition of placebo [24]. Although EGFR may not be beneficial for the survival of patients with gastric cancer, they were found to have significant predictive ability for the prognosis of patients [39].

Tyrosine kinase (TK) works downstream of EGFR and VEGFR in the same pathway [40]. Sunitinib inhibits cellular signaling by targeting multiple receptor tyrosine kinases including platelet-derived growth factor receptors (PDGF-Rs) and VEGFRs. However, according to this NMA, although not the worst, the performance of sunitinib was nor outstanding in either improving survival rate or controlling adverse events.

Hepatocyte growth factor receptor (HGFR) is encoded by c-MET gene and plays an important part in epithelial–mesenchymal transition and Onartuzumab belongs to this group. Previous research reported little effect of onartuzumab on improving survival rate, [41], and similar results were also observed in our network meta-analysis, with its effect on survival rate inferior to placebo and exhibited mediocre performance in reducing adverse events.

The alteration of mammalian target of rapamycin (mTOR) pathway is prevailing in tumor. In our result, everolimus, an mTOR inhibitor, was found to be efficacious in 1y-PFS and 2y-PFS. However, the result was denied by previous studies. Therefore, further clinical data was needed for the verification of our result.

Some limitation should be addressed before the application of our results. Firstly, the results can easily be diluted by the lack of evidence. For example, most of the treatments, including endostar, everolimus, matuzumab, onartuzumab, panitumumab and sunitinib were respectively covered by just one study and the number of patients involving in some treatments was relatively small Secondly, in addition to the variety of agents, the regimens of chemotherapy could also affect the prognosis of patients while In this NMA, we did not discriminate the chemotherapy that targeted drugs were combined with, which may add heterogeneity to this study. Moreover, alongside with gastric cancer, some patients with gastro-esophageal cancer were also included in our study, while may also cast doubt on the reliability of this NMA. Nonetheless, despite all these, this is the very first NMA comparing the efficacy and efficacy of different agents, and the selection criteria guaranteed the reliability of included studies.

In the current analysis, we evaluated the efficacy and adverse events of targets agents for gastric cancer. Ramucirumab was efficacious during one year to three years survival rate. However, high risk of hematological events should be noted during the application. Lapatinib was another extraordinary efficacious treatment in improving the overall survival condition, yet the severe adverse events made it not very recommended. Trastuzumab showed high efficacy in improving survival rate and was associated with relatively mild adverse events, therefore, it was recommended as the optimal targeted agent in clinical application. Meanwhile, physicians should consider the mutation of patients, the efficacy of agents, response rate, and side effects to select appropriate treatment.

## MATERIALS AND METHODS

### Study selection

We conducted our literature in PubMed, Embase, the Cochrane Library and Scopus. Literatures published between January 1st 2000 and October 1st 2016 were enrolled in our analysis. To start with, relevant studies were identified using key words “gastric cancer”, “chemotherapy”, “ramucirumab”, “bevacizumab”, “nimotuzumab”, “cetuximab”, “lapatinib”, “trastuzumab”, “endostar”, “everolimus”, “matuzumab”, “onartuzumab”, “panitumumab” and “sunitinib”. Irrelevant and duplicate articles were subsequently excluded by screening titles and abstracts. Two authors also reviewed the reference lists of enrolled articles for related study.

We analyzed the full text of retrieved articles. Studies in accordance with our inclusion criteria were enrolled for further analysis. Inclusion criteria: (1) study has to be a randomized clinical trial (RCT); (2) pathological diagnosis of gastric cancer should be confirmed in the study; (3) subjects should be aged from 18 to 80; (4) patients should be diagnosed with untreated advanced gastric cancer(5) target agents should be applied in combination with chemotherapy. Exclusion criteria: (1) treatment cannot form a network; (2) treatment lacked former chemotherapy; (3) treatment does not compare with others; (4) outcomes lacked sufficient information.

Moreover, all included articles were evaluated by Jadad scale [42]. Methods of studies were graded by randomization, blind method and withdrawal. Only studies with relatively high Jadad score were enrolled for further analysis.

### Data extraction

Two authors independently extracted related information from enrolled articles, including last name of first author, year of publication, country, follow-up duration (in month), sample size, average age of all subjects, targeted agents and their targets. Primary outcomes include 1 year overall survival (1y-OS), 2 year overall survival (2y-OS), 3 year overall survival (3y-OS), 1 year progression-free survival (1y-PFS), 2 year progression-free survival (2y-PFS) and 3 year progression-free survival (3y-PFS). OS was defined as the time from random diagnosis of gastric cancer to death of patients or the latest date known to be alive for censored patients. Progression-free survival (PFS) was defined as the time from diagnosis to first documented progression. Secondary outcomes are adverse events, including fatigue, anaemia, vomiting, diarrhoea, nausea and neutropenia. Discrepancies between two authors were resolved by a third author after discussion.

### Data analysis

In the current study, we performed a network meta-analysis to assess the outcomes of commonly used targeted agents in treating gastric cancer.

Our network meta-analysis is performed with a Bayesian model in WinBUGS (MRC Bio-statistics Unit, Cambridge, UK). Primary outcomes including 1y-OS, 2y-OS, 3y-OS, 1y-PFS, 2y-PFS and 3y-PFS were represented by hazard ratio (HR) with 95% corresponding credible interval (CrI); adverse events were represented by odds ratio (OR) with 95% corresponding CrI. Moreover, we calculated the surface under the cumulative ranking curve (SUCRA) to illustrate the rank probability of each agent for different outcomes based on the result of network meta-analysis.

## SUPPLEMENTARY MATERIALS FIGURES AND TABLES








