# Supplementary material for: The relative efficacy and safety of targeted agents used in combination with chemotherapy in treating patients with untreated advanced gastric cancer: a network meta-analysis
Source: Oncotarget. 2017 Mar 6;8(16):26959–68. doi: 10.18632/oncotarget.15923 (PMC5432310; doi:10.18632/oncotarget.15923)
Supplement: Supplementary file 1 [file oncotarget-08-26959-s001.pdf]

The relative efficacy and safety of targeted agents used in combination with chemotherapy in treating patients with untreated advanced gastric cancer: a network meta-analysis

Supplementary Materials

Supplementary Table 1: Main characteristics of included studies. See Supplementary_Table_1

Supplementary Table 2: Network meta-analysis results of overall survival and progression-free survival for gastric cancer therapy, represented by hazard ratio (HR) and 95% credible interval (CrI). See Supplementary_Table_2

Supplementary Table 3: Network meta-analysis results of adverse events for gastric cancer therapy, represented by odds ratio (OR) and 95% credible interval (CrI). See Supplementary_Table_3

Supplementary Table 4: Jadad scale

Author, Year	Randomization	Blinding	Withdrawal
Bang, 2010	2	0	1
Du, 2015	2	0	1
Fuchs, 2014	2	0	1
Lordick, 2013	2	0	1
Ohtsu, 2011	2	2	1
Satoh, 2015	2	2	1
Satoh, 2014	2	0	1
Shitara, 2016	2	0	1
Wilke, 2014	2	2	1
Xu, 2013	2	0	1
Yi, 2012	2	0	1
Casak, 2015	2	2	1
Hecht, 2016	2	2	1
Muro, 2016	2	0	1
Rao, 2010	2	2	1
Shan, 2016	2	2	1
Shen, 2015	2	2	1
Tebbutt, 2016	2	0	1
Xu, 2014	2	0	1
Shen, 2013	2	2	1
Ohtsu, 2013	2	2	1
Richards, 2013	2	0	1
Van Cutsem, 2013	2	0	1

The questions were as follows:
1. Was the study described as randomized?
2. Was the study described as double blind?
3. Was there a description of withdrawals and dropouts?
To receive the corresponding point, an article should describe the number of withdrawals and dropouts, in each of the study groups, and the underlying reasons.
Additional points were given if:
1. The method of randomization was described in the paper, and that method was appropriate. (1 extra point in randomization part)
2. The method of blinding was described, and it was appropriate. (1 extra point in blinding part)
Points would however be deducted if:
The method of randomization was described, but was inappropriate.
The method of blinding was described, but was inappropriate.
