# Supplementary material for: The relative efficacy and safety of targeted agents used in combination with chemotherapy in treating patients with untreated advanced gastric cancer: a network meta-analysis
Source: Oncotarget. 2017 Mar 6;8(16):26959–68. doi: 10.18632/oncotarget.15923 (PMC5432310; doi:10.18632/oncotarget.15923)
Supplement: Supplementary file 2 [file oncotarget-08-26959-s002.docx]

**Table 1. Main characteristics of included studies**

| **Author, Year** | **Country** | **Follow-up**  **(month)** | **Stage** | **Chemotherapy** | **Experiment group** | | | |  | **Control group** | | | **Outcomes** |
| --- | --- | --- | --- | --- | --- | --- | --- | --- | --- | --- | --- | --- | --- |
|  |  |  |  |  | **Size** | **Treatment** | **Target** | **Age** |  | **Size** | **Treatment** | **Age** |  |
| Bang 2010 | Korea | 34 | advanced gastric or gastro-oesophageal | CAP+CIS/FU+CIS | 294 | Trastuzumab | HER2 | 59.4 |  | 290 | Placebo | 58.5 | ①②③④⑤⑥⑦⑧ |
| Du 2015 | China | 60 | untreated unresectable or metastatic | S-1+CIS | 31 | Nimotuzumab | EGFR | 58 |  | 31 | Placebo | 31 | ①②③④⑤⑥⑦ |
| Fuchs 2014 | USA | 28 | advanced gastric or gastro-oesophageal | previous with pla and flu | 238 | Ramucirumab | VEGFR | 60 |  | 117 | Placebo | 60 | ①②④⑦⑧ |
| Lordick 2013 | Germany | 42 | advanced gastric or gastro-oesophageal | CAP+CIS | 455 | Cetuximab | EGFR | 60 |  | 449 | Placebo | 59 | ①②③④⑥⑦⑧ |
| Ohtsu 2011 | Japan | 24 | advanced gastric | CAP+CIS/FU+CIS | 387 | Bevacizumab | VEGF | 58 |  | 387 | Placebo | 59 | ①②③④⑥⑦ |
| Satoh 2015 | Japan | 20 | advanced gastric | IRI | 40 | Nimotuzumab | EGFR | 60 |  | 42 | Placebo | 63.5 | ①②③④⑤⑥⑦⑧ |
| Satoh 2014 | Japan | 45 | advanced gastric | PAC | 132 | Lapatinib | HER2 | 60.8 |  | 129 | Placebo | 60.4 | ①②③④⑤⑥⑦⑧ |
| Shitara 2016 | Japan | 28 | advanced gastric or gastro-oesophageal | PAC | 68 | Ramucirumab | VEGFR | 64 |  | 72 | Placebo | 64.5 | ①②④⑤⑥⑧ |
|  |  |  |  |  | 198 | Ramucirumab | VEGFR | 60 |  | 200 | Placebo | 61 | ①②④⑤⑥⑧ |
| Wilke 2014 | Germany | 28 | advanced gastric | PAC | 330 | Ramucirumab | VEGFR | 61 |  | 335 | Placebo | 61 | ①②③④⑤⑥⑦⑧ |
| Xu 2013 | China | 30 | advanced gastric | S-1+OXA | 80 | Endostar | VEGF | - |  | 85 | Placebo | - | ②③⑤⑥⑦ |
| Yi 2012 | Korea | 24 | unresectable or metastatic gastric cancer | DOC (previous with pla and flu) | 56 | Sunitinib | TKI | 54 |  | 49 | Placebo | 52 | ①②③④⑤⑥⑦ |
| Casak 2015 | USA | 30 | advanced or metastatic gastric or gastroesophageal junction | PAC | 267 | Ramucirumab | VEGFR | - |  | 241 | Placebo | - | ①②④⑤⑥⑦⑧ |
| Hecht 2016 | USA | 45 | advanced gastric or gastro-oesophageal | CAP+OXA | 249 | Lapatinib | HER2 | 61 |  | 238 | Placebo | 59 | ①②③④⑤⑥⑦ |
| Muro 2016 | Japan | 28 | advanced gastric or gastroesophageal junction | PAC (previous with pla and flu) | 109 | Ramucirumab | VEGFR | 62 |  | 114 | Placebo | 62 | ①②③④⑤⑥⑦⑧ |
|  |  |  |  |  | 221 | Ramucirumab | VEGFR | 60 |  | 221 | Placebo | 60 | ①②③④⑤⑥⑦⑧ |
| Rao 2010 | UK | 24 | advanced oesophago-gastric | CAP+CIS+EPI | 35 | Matuzumab | EGFR | 59 |  | 36 | Placebo | 64 | ①②③④⑤⑥⑦⑧ |
| Shan 2016 | USA | 17 | advanced gastric or gastroesophageal junction | FU+LV+OXA | 62 | Onartuzumab | HGRF | 58.5 |  | 61 | Placebo | 57 | ①②③④⑤⑥⑧ |
| Shen 2015 | China | 27 | locally advanced or metastatic gastric | CAP+CIS | 100 | Bevacizumab | VEGF | 54.2 |  | 102 | Placebo | 55.5 | ①②③④⑤⑥⑦ |
| Tebbutt 2016 | Australia | 18 | advanced oesophagogastric | DOC+FU | 37 | Panitumumab | EGFR | 64 |  | 39 | Placebo | 59 | ①②③④⑤⑧ |
| Xu 2014 | China | 15 | advanced gastric | CIS+CAP+FU | 17 | Nimotuzumab | EGFR | - |  | 17 | Placebo | - | ①② |
| Shen 2013 | China | 34 | advanced gastric or gastroesophageal junction | CAP+CIS/FU+CIS | 36 | Trastuzumab | HER2 | - |  | 48 | Placebo | - | ①②③④⑤⑥⑦⑧ |
| Ohtsu 2013 | Japan | 24 | advanced gastric | - | 439 | Everolimus | mTOR | 62 |  | 217 | Placebo | 62 | ①②③④⑤⑥⑦⑧ |
| Richards 2013 | USA | 36 | metastatic gastric or gastroesophageal | DOC+OXA (previous with FU+LV+RT) | 75 | Cetuximab | EGFR | 64 |  | 75 | Placebo | 61.7 | ①②⑤⑥⑧ |
| Van Cutsem 2013 | Belgium | 24 | Advanced gastric | CAP+CIS/FU+CIS | 387 | Bevacizumab | VEGF | - |  | 387 | Placebo | - | ①②④⑤⑥⑦⑧ |
