# Supplementary material for: The relative efficacy and safety of targeted agents used in combination with chemotherapy in treating patients with untreated advanced gastric cancer: a network meta-analysis
Source: Oncotarget. 2017 Mar 6;8(16):26959–68. doi: 10.18632/oncotarget.15923 (PMC5432310; doi:10.18632/oncotarget.15923)
Supplement: Supplementary file 3 [file oncotarget-08-26959-s003.docx]

**Table 2. Network meta-analysis results of overall survival and progression-free survival for gastric cancer therapy, represented by hazard ratio (HR) and 95% credible interval (CrI).**

| **1-OS** | **Placebo** | 0.72(0.63,0.82) | 1.06(0.88,1.29) | 0.70(0.55,0.89) | 0.78(0.64,0.94) | 1.54(1.14,2.08) | 1.19(0.92,1.54) | 1.03(0.78,1.35) | 1.03(0.78,1.36) | 0.58(0.51,0.66) | 0.76(0.59,0.98) | 0.67(0.51,0.88) | 0.79(0.52,1.19) | **1-PFS** |
| --- | --- | --- | --- | --- | --- | --- | --- | --- | --- | --- | --- | --- | --- | --- |
|  | 0.86(0.74,1.01) | **Bevacizumab** | 1.48(1.18,1.86) | 0.98(0.74,1.29) | 1.08(0.86,1.37) | 2.14(1.55,2.97) | 1.66(1.25,2.21) | 1.43(1.06,1.94) | 1.43(1.05,1.95) | 0.80(0.67,0.97) | 1.06(0.80,1.41) | 0.93(0.69,1.26) | 1.10(0.71,1.69) |  |
|  | 1.11(0.89,1.38) | 1.29(0.98,1.68) | **Cetuximab** | 0.66(0.48,0.90) | 0.73(0.56,0.96) | 1.45(1.02,2.06) | 1.12(0.81,1.54) | 0.97(0.69,1.35) | 0.97(0.69,1.36) | 0.54(0.43,0.68) | 0.71(0.52,0.98) | 0.63(0.45,0.88) | 0.74(0.47,1.17) |  |
|  | 0.89(0.68,1.17) | 1.03(0.75,1.41) | 0.80(0.57,1.14) | **Everolimus** | 1.11(0.82,1.52) | 2.20(1.49,3.23) | 1.70(1.20,2.42) | 1.47(1.02,2.12) | 1.47(1.02,2.13) | 0.82(0.62,1.09) | 1.09(0.76,1.54) | 0.96(0.66,1.38) | 1.13(0.70,1.82) |  |
|  | 0.81(0.65,1.02) | 0.94(0.72,1.24) | 0.73(0.54,1.00) | 0.91(0.64,1.30) | **Lapatinib** | 1.98(1.39,2.82) | 1.53(1.11,2.10) | 1.32(0.95,1.85) | 1.32(0.94,1.85) | 0.74(0.59,0.93) | 0.98(0.71,1.34) | 0.86(0.62,1.20) | 1.01(0.64,1.60) |  |
|  | 1.56(1.08,2.25) | 1.81(1.21,2.70) | 1.41(0.92,2.16) | 1.75(1.11,2.77) | 1.92(1.25,2.95) | **Matuzumab** | 0.77(0.52,1.15) | 0.67(0.45,1.00) | 0.67(0.44,1.01) | 0.38(0.27,0.52) | 0.49(0.33,0.73) | 0.44(0.29,0.65) | 0.51(0.31,0.85) |  |
|  | 0.94(0.79,1.12) | 1.09(0.86,1.38) | 0.85(0.64,1.12) | 1.06(0.76,1.47) | 1.16(0.87,1.54) | 0.60(0.40,0.91) | **Nimotuzumab** | 0.87(0.60,1.26) | 0.87(0.59,1.26) | 0.48(0.36,0.65) | 0.64(0.45,0.91) | 0.56(0.39,0.82) | 0.66(0.41,1.08) |  |
|  | 1.04(0.72,1.49) | 1.21(0.81,1.79) | 0.94(0.62,1.43) | 1.17(0.74,1.84) | 1.28(0.84,1.96) | 0.67(0.40,1.12) | 1.10(0.74,1.65) | **Onartuzumab** | 1.00(0.68,1.48) | 0.56(0.41,0.76) | 0.74(0.51,1.07) | 0.65(0.44,0.96) | 0.77(0.47,1.26) |  |
|  | 1.28(0.89,1.84) | 1.48(1.00,2.20) | 1.15(0.76,1.76) | 1.44(0.91,2.27) | 1.57(1.03,2.41) | 0.82(0.49,1.38) | 1.36(0.91,2.04) | 1.23(0.74,2.05) | **Panitumumab** | 0.56(0.41,0.76) | 0.74(0.51,1.08) | 0.65(0.44,0.96) | 0.77(0.47,1.26) |  |
|  | 0.73(0.65,0.82) | 0.85(0.70,1.03) | 0.66(0.52,0.85) | 0.82(0.61,1.11) | 0.90(0.70,1.16) | 0.47(0.32,0.69) | 0.78(0.63,0.96) | 0.71(0.48,1.03) | 0.57(0.39,0.84) | **Ramucirumab** | 1.32(0.99,1.75) | 1.16(0.86,1.57) | 1.37(0.89,2.11) |  |
|  | 0.95(0.70,1.30) | 1.10(0.78,1.56) | 0.86(0.59,1.25) | 1.07(0.70,1.62) | 1.17(0.80,1.72) | 0.61(0.38,0.99) | 1.01(0.71,1.44) | 0.91(0.57,1.47) | 0.74(0.46,1.20) | 1.29(0.93,1.81) | **Sunitinib** | 0.88(0.61,1.28) | 1.04(0.64,1.69) |  |
|  | 0.77(0.60,0.99) | 0.89(0.66,1.20) | 0.69(0.50,0.97) | 0.87(0.60,1.26) | 0.95(0.67,1.33) | 0.49(0.32,0.77) | 0.82(0.60,1.11) | 0.74(0.48,1.15) | 0.60(0.39,0.94) | 1.05(0.79,1.39) | 0.81(0.54,1.21) | **Trastuzumab** | 1.18(0.72,1.93) |  |
|  | - | - | - | - | - | - | - | - | - | - | - | - | **Endostar** |  |
| **2-OS** | **Placebo** | 0.76(0.69,0.83) | 1.07(0.97,1.19) | 0.66(0.56,0.78) | 0.78(0.68,0.88) | 1.13(0.63,2.02) | 2.14(1.19,3.84) | 1.08(0.71,1.64) | 0.61(0.56,0.66) | 0.77(0.52,1.15) | 0.68(0.59,0.78) | - | 0.54(0.44,0.66) | **2-PFS** |
|  | 0.85(0.77,0.95) | **Bevacizumab** | 1.41(1.23,1.62) | 0.87(0.72,1.05) | 1.02(0.87,1.20) | 1.49(0.83,2.68) | 2.82(1.56,5.09) | 1.42(0.93,2.18) | 0.80(0.71,0.91) | 1.01(0.67,1.53) | 0.90(0.76,1.06) | - | 0.71(0.57,0.89) |  |
|  | 1.05(0.94,1.18) | 1.23(1.05,1.44) | **Cetuximab** | 0.62(0.51,0.75) | 0.73(0.62,0.86) | 1.06(0.59,1.90) | 2.00(1.10,3.62) | 1.01(0.66,1.55) | 0.57(0.50,0.65) | 0.72(0.48,1.09) | 0.64(0.53,0.75) | - | 0.50(0.40,0.63) |  |
|  | 0.90(0.75,1.08) | 1.05(0.85,1.30) | 0.86(0.69,1.06) | **Everolimus** | 1.18(0.95,1.45) | 1.71(0.94,3.13) | 3.24(1.77,5.95) | 1.64(1.05,2.56) | 0.92(0.77,1.11) | 1.17(0.76,1.80) | 1.03(0.83,1.28) | - | 0.82(0.63,1.06) |  |
|  | 0.84(0.74,0.95) | 0.98(0.83,1.15) | 0.80(0.67,0.94) | 0.93(0.75,1.16) | **Lapatinib** | 1.45(0.80,2.64) | 2.75(1.51,5.01) | 1.39(0.90,2.15) | 0.79(0.67,0.91) | 0.99(0.65,1.51) | 0.88(0.72,1.06) | - | 0.70(0.55,0.88) |  |
|  | 1.02(0.61,1.70) | 1.19(0.71,2.01) | 0.97(0.57,1.64) | 1.13(0.66,1.95) | 1.22(0.72,2.07) | **Matuzumab** | 1.89(0.83,4.31) | 0.96(0.47,1.95) | 0.54(0.30,0.97) | 0.68(0.34,1.38) | 0.60(0.33,1.09) | - | 0.48(0.26,0.88) |  |
|  | 1.42(1.18,1.70) | 1.66(1.35,2.05) | 1.35(1.09,1.68) | 1.58(1.22,2.04) | 1.70(1.36,2.12) | 1.39(0.81,2.40) | **Nimotuzumab** | 0.50(0.25,1.03) | 0.29(0.16,0.51) | 0.36(0.18,0.73) | 0.32(0.17,0.58) | - | 0.25(0.14,0.47) |  |
|  | 1.06(0.64,1.75) | 1.24(0.74,2.07) | 1.01(0.60,1.69) | 1.18(0.69,2.01) | 1.27(0.76,2.13) | 1.04(0.51,2.13) | 0.75(0.44,1.27) | **Onartuzumab** | 0.56(0.37,0.86) | 0.71(0.40,1.27) | 0.63(0.41,0.98) | - | 0.50(0.31,0.79) |  |
|  | 0.80(0.73,0.87) | 0.93(0.82,1.06) | 0.76(0.66,0.87) | 0.88(0.72,1.08) | 0.95(0.82,1.11) | 0.78(0.46,1.31) | 0.56(0.46,0.69) | 0.75(0.45,1.25) | **Ramucirumab** | 1.26(0.84,1.90) | 1.11(0.95,1.31) | - | 0.89(0.71,1.10) |  |
|  | 0.94(0.60,1.48) | 1.10(0.69,1.75) | 0.90(0.56,1.43) | 1.04(0.64,1.70) | 1.12(0.70,1.80) | 0.92(0.46,1.83) | 0.66(0.41,1.08) | 0.89(0.45,1.75) | 1.18(0.74,1.88) | **Sunitinib** | 0.88(0.58,1.35) | - | 0.70(0.45,1.10) |  |
|  | 0.75(0.66,0.87) | 0.88(0.74,1.05) | 0.72(0.60,0.86) | 0.84(0.67,1.05) | 0.90(0.75,1.09) | 0.74(0.44,1.26) | 0.53(0.42,0.67) | 0.71(0.42,1.20) | 0.95(0.81,1.12) | 0.80(0.50,1.29) | **Trastuzumab** | - | 0.79(0.62,1.02) |  |
|  | 1.10(0.89,1.35) | 1.29(1.02,1.62) | 1.05(0.83,1.33) | 1.22(0.93,1.61) | 1.32(1.03,1.68) | 1.08(0.62,1.88) | 0.77(0.59,1.02) | 1.04(0.60,1.79) | 1.38(1.10,1.73) | 1.17(0.71,1.93) | 1.46(1.13,1.87) | **Panitumumab** | - |  |
|  | - | - | - | - | - | - | - | - | - | - | - | - | **Endostar** |  |
| **3-OS** | **Placebo** | 0.65(0.56,0.76) | 1.08(0.95,1.22) | 0.48(0.41,0.57) | 0.81(0.71,0.92) | - | 0.48(0.38,0.62) | 0.71(0.60,0.84) | **3-PFS** |  |  |  |  |  |
|  | 0.88(0.74,1.04) | **Bevacizumab** | 1.66(1.36,2.02) | 0.74(0.59,0.92) | 1.25(1.02,1.52) | - | 0.74(0.55,1.00) | 1.09(0.87,1.37) |  |  |  |  |  |  |
|  | 1.06(0.96,1.17) | 1.20(0.99,1.47) | **Cetuximab** | 0.44(0.36,0.55) | 0.75(0.63,0.90) | - | 0.45(0.34,0.59) | 0.66(0.53,0.81) |  |  |  |  |  |  |
|  | - | - | - | **Endostar** | 1.69(1.37,2.08) | - | 1.01(0.75,1.36) | 1.47(1.16,1.87) |  |  |  |  |  |  |
|  | 0.87(0.79,0.97) | 0.99(0.81,1.22) | 0.83(0.71,0.96) | - | **Lapatinib** | - | 0.60(0.45,0.79) | 0.87(0.70,1.08) |  |  |  |  |  |  |
|  | 1.24(0.85,1.80) | 1.41(0.94,2.13) | 1.17(0.80,1.72) | - | 1.42(0.96,2.09) | **Nimotuzumab** | - | - |  |  |  |  |  |  |
|  | - | - | - | - | - | - | **Ramucirumab** | - |  |  |  |  |  |  |
|  | 0.74(0.61,0.90) | 0.84(0.65,1.09) | 0.70(0.56,0.87) | - | 0.84(0.67,1.05) | 0.59(0.39,0.91) | - | **Trastuzumab** |  |  |  |  |  |  |

Note that the upper half of a table is transposed, thus row treatments are compared against column treatments (whereas in the lower half, column treatments are compared against row treatments).
