# Supplementary material for: The relative efficacy and safety of targeted agents used in combination with chemotherapy in treating patients with untreated advanced gastric cancer: a network meta-analysis
Source: Oncotarget. 2017 Mar 6;8(16):26959–68. doi: 10.18632/oncotarget.15923 (PMC5432310; doi:10.18632/oncotarget.15923)
Supplement: Supplementary file 4 [file oncotarget-08-26959-s004.docx]

**Table 3. Network meta-analysis results of adverse events for gastric cancer therapy, represented by odds ratio (OR) and 95% credible interval (CrI)**

| **Fatigue** | **Placebo** | 0.80(0.38,1.68) | 1.35(0.64,2.92) | 1.46(0.65,3.35) | 1.15(0.13,11.70) | 1.58(0.42,5.87) | 0.75(0.50,1.09) | 1.08(0.51,2.20) | - | - | 0.77(0.41,1.48) | 1.48(0.26,8.50) | 1.06(0.25,4.39) | **Anaemia** |
| --- | --- | --- | --- | --- | --- | --- | --- | --- | --- | --- | --- | --- | --- | --- |
|  | 1.43(0.59,3.60) | **Cetuximab** | 1.68(0.58,4.95) | 1.82(0.61,5.70) | 1.45(0.14,16.12) | 1.97(0.43,8.76) | 0.94(0.40,2.14) | 1.35(0.45,3.71) | - | - | 0.96(0.37,2.61) | 1.82(0.28,12.30) | 1.34(0.26,6.55) |  |
|  | 1.60(0.47,5.70) | 1.12(0.24,5.26) | **Everolimus** | 1.08(0.36,3.35) | 0.85(0.08,9.49) | 1.16(0.26,5.21) | 0.55(0.23,1.28) | 0.81(0.27,2.23) | - | - | 0.57(0.21,1.58) | 1.08(0.16,8.00) | 0.79(0.16,3.90) |  |
|  | 8.94(0.93,290.03) | 6.30(0.55,221.41) | 5.70(0.41,219.20) | **Lapatinib** | 0.78(0.08,9.21) | 1.07(0.21,4.95) | 0.51(0.20,1.23) | 0.75(0.23,2.18) | - | - | 0.53(0.19,1.51) | 0.99(0.15,7.10) | 0.73(0.14,3.71) |  |
|  | 1.54(0.31,8.33) | 1.07(0.18,7.10) | 0.95(0.13,7.77) | 0.17(0.00,2.97) | **Matuzumab** | 1.34(0.09,18.73) | 0.64(0.06,6.11) | 0.92(0.08,9.78) | - | - | 0.67(0.06,6.75) | 1.25(0.06,24.78) | 0.92(0.06,13.07) |  |
|  | 1.08(0.14,8.17) | 0.76(0.08,6.75) | 0.68(0.06,7.17) | 0.11(0.00,2.56) | 0.70(0.05,9.49) | **Nimotuzumab** | 0.48(0.12,1.90) | 0.68(0.15,3.06) | - | - | 0.49(0.12,2.16) | 0.95(0.11,8.50) | 0.68(0.10,4.62) |  |
|  | 1.90(1.02,2.94) | 1.32(0.42,3.35) | 1.17(0.27,4.06) | 0.21(0.01,2.05) | 1.22(0.20,6.30) | 1.72(0.21,14.15) | **Ramucirumab** | 1.45(0.62,3.25) | - | - | 1.03(0.50,2.25) | 1.95(0.33,12.68) | 1.42(0.33,6.23) |  |
|  | 1.95(0.60,7.03) | 1.36(0.31,6.36) | 1.20(0.22,7.32) | 0.21(0.01,3.00) | 1.26(0.16,9.78) | 1.80(0.18,20.29) | 1.03(0.30,4.48) | **Trastuzumab** | - | - | 0.71(0.28,2.01) | 1.36(0.21,8.94) | 0.99(0.19,5.05) |  |
|  | 0.61(0.17,2.14) | 0.43(0.09,1.95) | 0.38(0.06,2.18) | 0.07(0.00,0.89) | 0.39(0.05,2.94) | 0.55(0.05,6.42) | 0.32(0.09,1.38) | 0.32(0.05,1.70) | **Onartuzumab** | - | - | - | - |  |
|  | 1.99(0.46,9.03) | 1.40(0.24,7.92) | 1.23(0.18,8.67) | 0.21(0.01,3.39) | 1.30(0.14,11.36) | 1.82(0.15,24.05) | 1.06(0.24,5.53) | 1.02(0.14,6.82) | 3.25(0.48,23.10) | **Panitumumab** | - | - | - |  |
|  | - | - | - | - | - | - | - | - | - | - | **Bevacizumab** | 1.90(0.30,12.06) | 1.38(0.28,6.42) |  |
|  | - | - | - | - | - | - | - | - | - | - | - | **Endostar** | 0.73(0.07,6.75) |  |
|  | - | - | - | - | - | - | - | - | - | - | - | - | **Sunitinib** |  |
| **Diarrhoea** | **Placebo** | 1.17(0.57,2.69) | 0.90(0.32,2.46) | 0.71(0.21,2.41) | 1.31(0.51,3.39) | 1.15(0.03,46.99) | 1.48(0.26,9.12) | 1.03(0.34,3.19) | 1.08(0.23,4.85) | 0.75(0.43,1.28) | 0.92(0.02,40.45) | 1.06(0.46,2.72) | - | **Vomiting** |
|  | 0.61(0.07,4.57) | **Bevacizumab** | 0.77(0.20,2.56) | 0.61(0.14,2.46) | 1.12(0.31,3.60) | 0.96(0.02,41.26) | 1.25(0.18,8.85) | 0.88(0.21,3.25) | 0.91(0.16,4.76) | 0.63(0.23,1.55) | 0.77(0.02,35.16) | 0.90(0.28,2.94) | - |  |
|  | 1.62(0.46,5.99) | 2.66(0.25,35.16) | **Cetuximab** | 0.79(0.16,3.90) | 1.48(0.36,5.81) | 1.27(0.03,63.43) | 1.65(0.22,13.07) | 1.16(0.26,5.26) | 1.20(0.19,7.32) | 0.84(0.26,2.61) | 1.02(0.02,49.40) | 1.17(0.33,4.90) | - |  |
|  | 4.31(0.93,38.09) | 7.54(0.55,149.90) | 2.77(0.36,32.79) | **Everolimus** | 1.86(0.39,8.50) | 1.58(0.03,81.45) | 2.08(0.24,17.81) | 1.45(0.28,7.54) | 1.51(0.21,10.28) | 1.04(0.27,3.86) | 1.30(0.03,63.43) | 1.49(0.35,7.17) | - |  |
|  | 5.70(2.48,14.73) | 9.30(1.03,103.54) | 3.49(0.77,16.78) | 1.31(0.13,7.85) | **Lapatinib** | 0.88(0.02,41.68) | 1.11(0.16,9.03) | 0.78(0.18,3.53) | 0.81(0.14,4.90) | 0.57(0.19,1.70) | 0.70(0.02,33.45) | 0.81(0.23,3.22) | - |  |
|  | 0.98(0.08,11.02) | 1.60(0.06,39.25) | 0.60(0.03,8.76) | 0.22(0.01,4.06) | 0.17(0.01,2.14) | **Matuzumab** | 1.30(0.02,82.27) | 0.92(0.02,43.82) | 0.93(0.02,48.42) | 0.65(0.02,27.94) | 0.78(0.01,139.77) | 0.93(0.02,42.10) | - |  |
|  | 2.41(0.36,25.53) | 3.97(0.25,103.54) | 1.49(0.16,22.20) | 0.55(0.03,9.78) | 0.42(0.05,5.16) | 2.56(0.12,79.04) | **Nimotuzumab** | 0.70(0.08,5.58) | 0.73(0.07,7.39) | 0.50(0.08,3.13) | 0.63(0.01,38.09) | 0.72(0.10,5.21) | - |  |
|  | 1.38(0.45,4.10) | 2.27(0.22,26.58) | 0.85(0.16,4.53) | 0.32(0.03,2.08) | 0.24(0.06,0.92) | 1.40(0.10,23.34) | 0.58(0.04,5.00) | **Onartuzumab** | 1.04(0.15,6.69) | 0.72(0.21,2.46) | 0.89(0.02,43.82) | 1.02(0.26,4.53) | - |  |
|  | 1.84(0.43,7.85) | 3.00(0.25,46.06) | 1.14(0.16,7.92) | 0.41(0.03,3.49) | 0.32(0.06,1.72) | 1.86(0.12,40.04) | 0.75(0.04,8.17) | 1.34(0.22,8.17) | **Panitumumab** | 0.69(0.14,3.60) | 0.85(0.02,46.99) | 0.99(0.18,6.17) | - |  |
|  | 2.39(1.27,4.76) | 3.94(0.47,38.86) | 1.48(0.34,6.05) | 0.54(0.06,3.00) | 0.42(0.14,1.22) | 2.46(0.20,33.78) | 0.99(0.09,7.39) | 1.72(0.50,6.36) | 1.31(0.26,6.55) | **Ramucirumab** | 1.25(0.03,54.60) | 1.43(0.52,4.31) | - |  |
|  | 6.75(0.84,145.47) | 11.70(0.63,441.42) | 4.31(0.35,115.58) | 1.52(0.08,45.15) | 1.20(0.12,27.39) | 7.61(0.26,343.78) | 2.83(0.13,103.54) | 5.00(0.47,135.64) | 3.82(0.30,111.05) | 2.86(0.30,67.36) | **Sunitinib** | 1.17(0.02,51.42) | - |  |
|  | 2.64(1.02,7.24) | 4.31(0.46,47.94) | 1.62(0.33,8.17) | 0.59(0.05,4.01) | 0.46(0.12,1.67) | 2.69(0.20,40.85) | 1.08(0.08,9.21) | 1.88(0.45,8.67) | 1.43(0.24,8.41) | 1.11(0.34,3.56) | 0.39(0.01,3.78) | **Trastuzumab** | - |  |
|  | 1.25(0.02,46.53) | 1.93(0.02,138.38) | 0.75(0.01,33.45) | 0.27(0.00,13.33) | 0.21(0.00,8.85) | 1.27(0.01,125.21) | 0.47(0.01,32.79) | 0.90(0.01,41.26) | 0.66(0.01,34.81) | 0.51(0.01,20.70) | 0.16(0.00,12.43) | 0.47(0.01,19.69) | **Endostar** |  |
| **Neutropenia** | **Placebo** | 0.84(0.40,2.23) | 0.73(0.25,2.14) | 0.93(0.22,3.78) | 1.03(0.29,3.67) | 2.27(0.78,6.55) | 6.42(0.61,177.68) | 1.86(0.43,8.33) | 1.17(0.36,3.86) | 0.74(0.27,1.95) | 0.87(0.02,35.87) | 1.17(0.48,3.19) | 1.97(0.49,8.41) | **Nausea** |
|  | 0.92(0.48,1.68) | **Bevacizumab** | 0.87(0.20,3.03) | 1.08(0.19,5.21) | 1.21(0.24,5.10) | 2.64(0.62,9.68) | 7.46(0.59,221.41) | 2.14(0.39,11.94) | 1.38(0.29,5.42) | 0.87(0.21,2.92) | 0.99(0.02,43.82) | 1.39(0.37,4.66) | 2.32(0.41,11.47) |  |
|  | 0.74(0.41,1.39) | 0.80(0.35,2.05) | **Cetuximab** | 1.27(0.21,7.17) | 1.40(0.26,7.32) | 3.10(0.68,13.87) | 8.94(0.67,275.89) | 2.53(0.41,15.80) | 1.60(0.32,7.92) | 1.01(0.23,4.22) | 1.17(0.03,55.70) | 1.60(0.40,7.17) | 2.69(0.47,16.12) |  |
|  | 1.00(0.36,2.86) | 1.08(0.33,3.74) | 1.35(0.40,4.44) | **Endostar** | 1.11(0.17,7.61) | 2.44(0.42,14.59) | 7.10(0.47,239.85) | 1.99(0.25,15.96) | 1.25(0.21,8.17) | 0.79(0.15,4.62) | 0.92(0.02,48.91) | 1.27(0.25,7.32) | 2.12(0.29,16.44) |  |
|  | 12.30(1.82,350.72) | 13.60(1.80,399.41) | 16.61(2.20,497.70) | 12.55(1.42,407.48) | **Everolimus** | 2.23(0.42,11.59) | 6.36(0.43,225.88) | 1.80(0.28,13.07) | 1.14(0.20,6.55) | 0.72(0.14,3.60) | 0.86(0.02,42.95) | 1.14(0.24,5.93) | 1.92(0.29,13.20) |  |
|  | 3.60(1.77,8.50) | 3.90(1.57,11.82) | 4.85(1.92,13.60) | 3.63(1.04,14.15) | 0.29(0.01,2.41) | **Lapatinib** | 2.89(0.22,88.23) | 0.82(0.13,5.16) | 0.52(0.11,2.59) | 0.33(0.07,1.40) | 0.39(0.01,17.81) | 0.52(0.13,2.27) | 0.88(0.15,5.21) |  |
|  | 1.16(0.35,3.94) | 1.27(0.34,5.10) | 1.57(0.41,5.93) | 1.16(0.24,5.75) | 0.09(0.00,0.96) | 0.32(0.07,1.30) | **Matuzumab** | 0.28(0.01,4.71) | 0.18(0.01,2.59) | 0.11(0.00,1.49) | 0.13(0.00,11.02) | 0.18(0.01,2.39) | 0.30(0.01,5.10) |  |
|  | 1.54(0.58,4.44) | 1.68(0.54,5.81) | 2.08(0.66,6.89) | 1.55(0.37,6.89) | 0.12(0.00,1.14) | 0.42(0.12,1.51) | 1.32(0.28,6.55) | **Nimotuzumab** | 0.64(0.09,4.22) | 0.39(0.07,2.34) | 0.47(0.01,22.87) | 0.64(0.11,3.63) | 1.06(0.14,8.00) |  |
|  | 1.65(0.60,4.57) | 1.77(0.55,6.05) | 2.23(0.68,7.10) | 1.63(0.38,6.89) | 0.13(0.00,1.17) | 0.45(0.12,1.55) | 1.42(0.29,6.69) | 1.06(0.25,4.26) | **Onartuzumab** | 0.63(0.13,2.89) | 0.75(0.02,36.23) | 1.00(0.23,4.81) | 1.68(0.26,10.38) |  |
|  | 2.89(2.08,4.22) | 3.10(1.60,6.75) | 3.90(1.92,7.85) | 2.89(0.98,8.76) | 0.23(0.01,1.65) | 0.80(0.32,1.77) | 2.48(0.70,8.67) | 1.88(0.62,5.26) | 1.75(0.61,5.21) | **Ramucirumab** | 1.19(0.03,56.26) | 1.60(0.42,6.42) | 2.69(0.47,15.64) |  |
|  | 1.86(0.61,5.93) | 1.99(0.57,7.69) | 2.48(0.68,9.21) | 1.82(0.39,8.85) | 0.15(0.00,1.42) | 0.51(0.12,1.97) | 1.57(0.31,8.58) | 1.20(0.26,5.37) | 1.12(0.25,5.21) | 0.64(0.19,2.12) | **Sunitinib** | 1.35(0.03,60.95) | 2.23(0.05,115.58) |  |
|  | 0.99(0.54,2.08) | 1.06(0.47,3.00) | 1.34(0.57,3.46) | 0.99(0.30,3.60) | 0.08(0.00,0.63) | 0.28(0.10,0.75) | 0.86(0.22,3.49) | 0.64(0.19,2.16) | 0.61(0.19,2.14) | 0.34(0.17,0.76) | 0.54(0.15,2.10) | **Trastuzumab** | 1.68(0.30,9.12) |  |
|  | - | - | - | - | - | - | - | - | - | - | - | - | **Panitumumab** |  |

Note that the upper half of a table is transposed, thus row treatments are compared against column treatments (whereas in the lower half, column treatments are compared against row treatments).
